# Supplementary material for: Funneliformis mosseae Improves Growth and Nutrient Accumulation in Wheat by Facilitating Soil Nutrient Uptake under Elevated CO2 at Daytime, Not Nighttime
Source: J Fungi (Basel). 2021 Jun 7;7(6):458. doi: 10.3390/jof7060458 (PMC8229587; doi:10.3390/jof7060458)
Supplement: Supplementary file 1 [file jof-07-00458-s001.zip › jof-1234363-supplementary.pdf]

Supplementary material

Funneliformis mosseae improves growth and nutrient accumulation in wheat by facilitating soil nutrient uptake under elevated CO_2 at daytime, not nighttime

Songmei Shi[1,2,†], Xie Luo[1,2,†], Miao Wen[1,2], Xingshui Dong[1,2], Sharifullah Sharifi[1,2], Deti Xie[1,2] and Xinhua He[1,2,3,*]

[1] Centre of Excellence for Soil Biology, College of Resources and Environment, and Chongqing Key Laboratory of Plant Resource Conservation and Germplasm Innovation, School of Life Sciences, Southwest University, Chongqing 400716, China; shismei@email.swu.edu.cn (S.S.); luox123@email.swu.edu.cn (X.L.); wenmiao77@163.com (W.M.); xingshuid@outlook.com (X.D.); nsharifullah@gmail.com (S.S.); xdt@swu.edu.cn (X.D.); xinhua.he@uwa.edu.au (X.H.)

[2] National Base of International S&T Collaboration on Water Environmental Monitoring and Simulation in Three Gorges Reservoir Region, Chongqing 400716, China

[3] School of Biological Sciences, University of Western Australia, Perth, WA 6009, Australia

† These two authors contributed to this paper equally

* Correspondence: xinhua.he@uwa.edu.au; Tel.: 86-18723289058

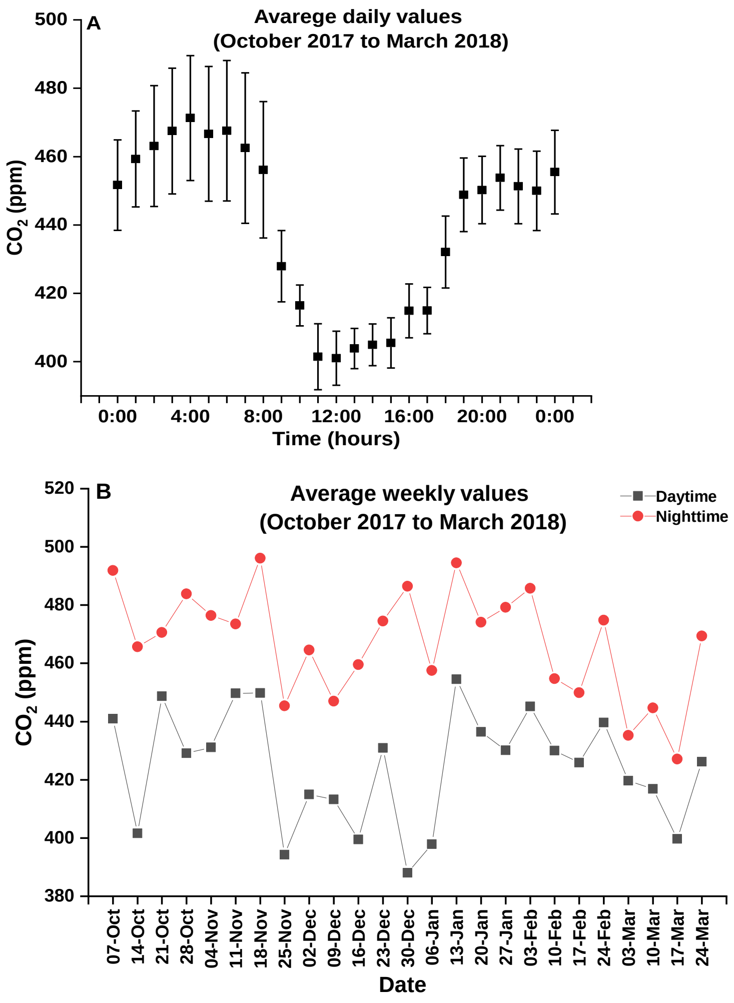

Figure S1. Average daily (A, 417±16/463±27 ppm, means ± SD, n =175, daytime (7:00 am-19:00 pm)/nighttime (19:00 pm-7:00 am)) and weekly (B, 421±19/467±18 ppm, means ± SD, n = 25, day-time/nighttime) variations of atmospheric CO_2 concentrations between October 2017 and March 2018 in the National Monitoring Base for Purple Soil Fertility and Fertilizer Efficiency (29°48′N, 106°24′E, 266.3m above the sea level) on the campus of Southwest University, Chongqing, China. The atmosphere CO_2 concentrations were monitored by an auto-controlled facility (DSS-QZD, Qingdao Shengsen Institute of Science and Technology, Shandong, China).

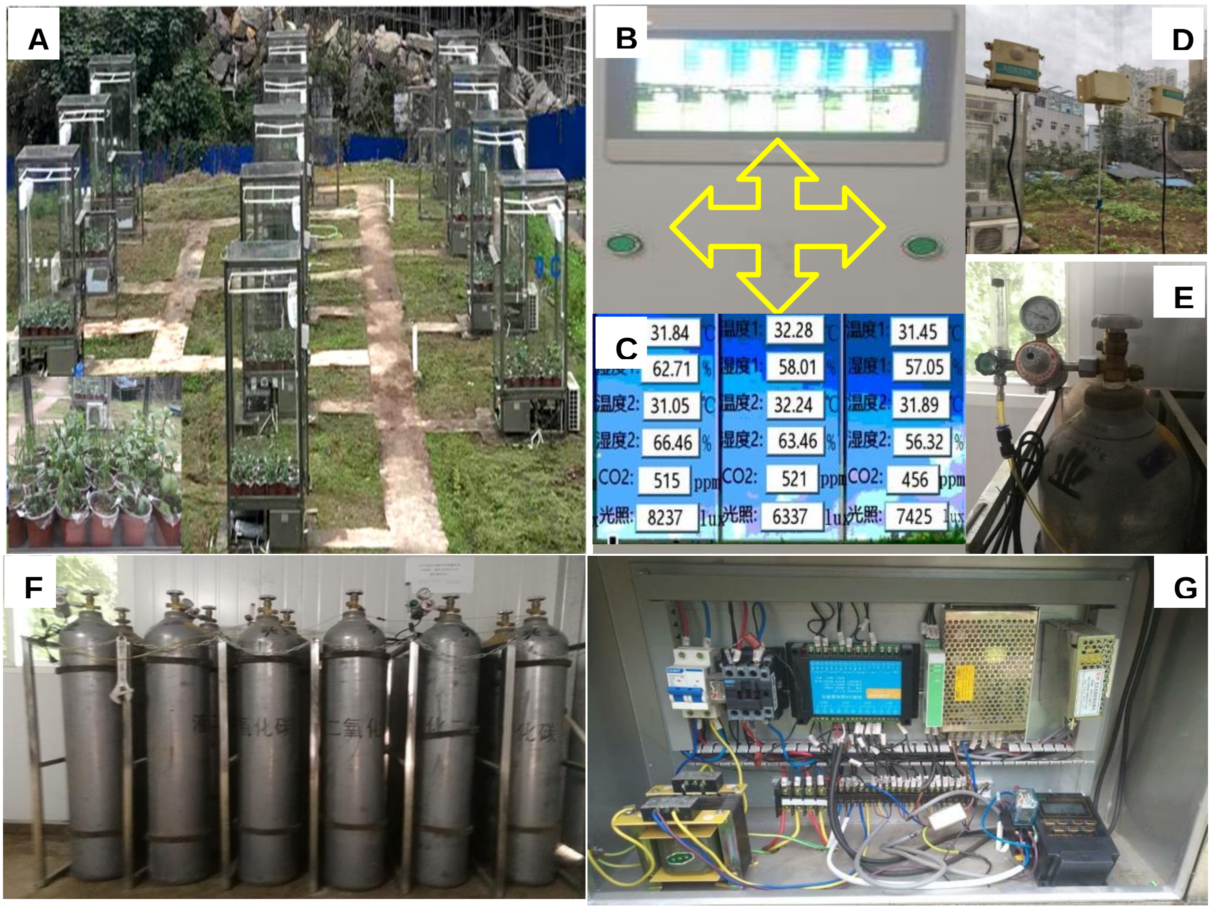

Figure S2. CO_2 auto-controlling facility (A) overview of environmentally controlled growth chambers, (B-C) CO_2 digital control screen, (D) the humidity, temperature, light and CO_2 sensors, (E-F) CO_2 gas cylinder, (G) the control system

A total of 12 growth environmentally controlled glass-made chambers were constructed in 2016 at the National Monitoring Station of Soil Fertility and Fertilizer Efficiency on Purple Soils, which is located on the Southwest University Campus, the Beibei District (29°48′N, 106°24′E, 266.3 m above the sea level), Chongqing, China. The temperatures and humidity inside the growth chambers were also automatically maintained as the same as those outside the chambers. The temperature, humidity, light and CO_2 concentration had been monitored every 6 second by a CO_2 auto-controlling facility. The plants grown in pots inside chambers had the same growth conditions (nitrogen-fertilization, temperature, light intensity, etc.), except CO_2 concentrations: ambient CO_2 (ACO_2, 410 ppm daytime/460 ppm nighttime), daytime eCO_2 only ($DeCO_2$, 550 ppm/460 ppm), nighttime eCO_2 only ($NeCO_2$, 410ppm/610ppm), and continuous daytime/nighttime eCO_2 ($D+NeCO_2$, 550 ppm/610 ppm). The respective daytime and nighttime eCO_2 concentrations were thus increased by ~33.33% of the ACO_2 treatment.
